# Phenolic Profile of *Potentilla anserina* L. (Rosaceae) Herb of Siberian Origin and Development of a Rapid Method for Simultaneous Determination of Major Phenolics in *P. anserina* Pharmaceutical Products by Microcolumn RP-HPLC-UV

**DOI:** 10.3390/molecules20010224

**Published:** 2014-12-24

**Authors:** Daniil N. Olennikov, Nina I. Kashchenko, Nadezhda K. Chirikova, Sargylana S. Kuz’mina

**Affiliations:** 1 Institute of General and Experimental Biology, Siberian Division, Russian Academy of Science, Sakh’yanovoy str., 6, Ulan-Ude 670047, Russia; E-Mail: ninkk@mail.ru; 2 Department of Biochemistry and Biotechnology, North-Eastern Federal University, 58 Belinsky Str., Yakutsk 677-027, Russia; E-Mails: hofnung@mail.ru (N.K.C.); sskuzmina@bk.ru (S.S.K.)

**Keywords:** *Potentilla anserina*, Rosaceae, microcolumn RP-HPLC-UV, agrimoniin, flavonoids, quality control

## Abstract

A chemical study of *Potentilla anserina* L. herb (Rosaceae) of Siberian origin led to the isolation of 17 compounds. Three ellagitannins—potentillin, agrimonic acid A and B—are reported for the first time in this species. With a view to rapid quantitative analysis, a new method was developed for simultaneous determination of major phenolic compounds in *P. anserina*, including caffeic acid, myricetin-3-*O*-glucuronide, agrimoniin, ellagic acid, miquelianin, isorhamnetin-3-*O*-glucuronide, and kaempferol-3-*O*-rhamnoside. The quantitative determination was conducted by microcolumn reversed phase high-performance liquid chromatography with UV detection. Separation was performed using a ProntoSIL-120-5-C18 AQ column (60 mm × 1 mm × 5 μm) with six-step gradient elution of aqueous 0.2 М LiClO_4_ in 0.006 M HClO_4_ and acetonitrile as mobile phases. The components were quantified by HPLC-UV at 270 nm. All calibration curves showed good linearity (*r*^2^ > 0.999) within test ranges. The reproducibility was evaluated by intra- and inter-day assays, and RSD values were less than 2.8%. The recoveries were between 97.15 and 102.38%. The limits of detection ranged from 0.21 to 1.94 μg/mL, and limits of quantification ranged from 0.65 to 5.88 μg/mL, respectively. Various solvents, extraction methods, temperatures, and times were evaluated to obtain the best extraction efficiency. The developed method was successfully applied for the analysis of selected pharmaceutical products: 12 batches of *P. anserina* herb collected from three Siberian regions (Yakutia, Buryatia, Irkutsk), two commercial samples of *P. anserina* herb, and some preparations (liquid extract, tincture, decoction, infusion, and dry extract).

## 1. Introduction

*Potentilla anserina* L. (silverweed) is a low-growing herbaceous plant of the Rosaceae family. It is found in the temperate zone around the globe and most commonly on sandy or rocky clay soils in Europe, Siberia, the Far East, and Central Asia. Remedies containing *P. anserina* total herb are widely used in various folk and medical systems, particularly in traditional Tibetan medicine and folk medicine. In Tibetan medicine, *P. anserina* herb is used for infectious diarrhea with fever, while its roots and rhizomes are applied as an antiseptic remedy [1,2]. In Yakutian medicine, the tincture from *P. anserina* flowers in vodka is used as an antidiarrheal remedy; the water decoction from flowers and leaves is applied for kidney and liver diseases, respectively. The gruel from the fresh leaves is used as a wound-healing agent [3]. In Chinese medicine, the whole plant is applied for the treatment of hematemesis [4]. Traditionally, Mongolian arats (cattle-breeders) applied *P. anserina* as an astringent and bactericidal remedy for treatment of enterocolitis and dyspepsia [5].

As a result of phytochemical investigations, the presence of the following compounds in the herb of *P. anserina* was established: catechins (catechin, gallocatechin [6], epigallocatechin, epicatechin, ellagic acid [7]); ellagitannins (agrimoniin, pedunculagin [8]); phenolic acids (gallic, *p*-hydroxybenzoic, vanillic, gentizic, *p*-hydroxyphenylacetic, *p*-coumaric, caffeic, ferulic [9], chlorogenic acids [10]); coumarins (umbelliferon, scopoletin [11]); flavonoids (myricetin [12], myricetin-3-*O*-β-d-glucuronide, myricitrin, isorhamnetin-3-*O*-β-d-glucuronide, isoquercitrin, reynoutrin, quercitrin, quercetin-3-*O*-β-d-sambubioside, miquelianin, astragalin, tiliroside, 8-methoxykaempferol-3-*O*-sophoroside [6], rutin, nicotoflorin, linarin [10]); isoflavones (genistein [10]); pyrones (2-pyrone-4,6-dicarboxylic acid [13]); aliphatic compounds (nonacosane, ceryl alcohol, arachidic, cerotic, palmitic, myristic, linoleic, linolenic and oleic acids [14]); and polysaccharides [15]. According to known data about the chemical constituents of *P. anserina*, the occurrence of different classes of phenolic compounds was previously reported for plant material collected in Europe (Poland, Germany, Italy, Ukraine) [6,7,8,9,10,11,12,13]. There is currently no information about the chemical composition of *P. anserina* growing in Siberia.

A great deal of pharmacological studies (*in vitro*,* in vivo*, clinical trials) have generally confirmed the traditional use of *P. anserina* and its extracts from aerial and/or underground parts for cardioprotective [16,17], hepatoprotective [18], spasmolytic [19], immunomodulatory [20], antiviral [21], antimutagenic [22,23], antimicrobial [24], antiradical [25], antihypoxic [26,27], and anti-inflammatory [28] remedies. Phenolic compounds are considered a biologically active group of metabolites possessing anti-inflammatory [28], antioxidant, and antibacterial activity [25] of *P. anserina* formulations.

Given the importance of quantitative analysis of the main phenolic components in *P. anserina* herb, some authors have suggested different variants of chromatographic analysis applied to HPLC assay, *viz.* HPLC-UV and HPLC-DAD (Table 1). In all of the proposed methods, reversed-phase sorbents and gradient elution were used for the separation of different classes of *P. anserina* metabolites. The known HPLC techniques used in quantitative analysis of phenolic compounds used NanoLC-Orbitrap-MS [10] and UHPLC-DAD-MS/MS [28]. All mentioned methods were purposed for simultaneous determination of flavonoids and phenylpropanoids [29], tannins and catechins [8], or flavonoids and catechins [7]. There is no HPLC technique allowing one to analyze the quantitative content of the major phenolic groups in *P. anserina* herb: ellagitannins, flavonoids and phenylpropanoids.

In the last decade, miniaturized separation techniques have become greatly popular in pharmaceutical analysis. Miniaturized separation methods are increasingly utilized in all processes of drug discovery as well as the quality control of pharmaceutical preparation [30]. The very low sample required per analysis, reduced analysis time, and the concern about environmental pollution have pushed the use of miniaturized techniques. The range of analysis time in the known variants of HPLC assays of *P. anserina* was from 32 min to 120 min. In order to reduce the analysis time, we examined the possibility of developing a rapid and validated method for simultaneous determination of major phenolics in *P. anserina* herb by microcolumn RP-HPLC-UV. Moreover, we realized chemical examination of *P. anserina* herb of the Siberian origin and some *P. anserina* pharmaceutical products.

## 2. Results and Discussion

### 2.1. Phenolic Profile of P. anserina Growing in Siberia

**Figure 1:**
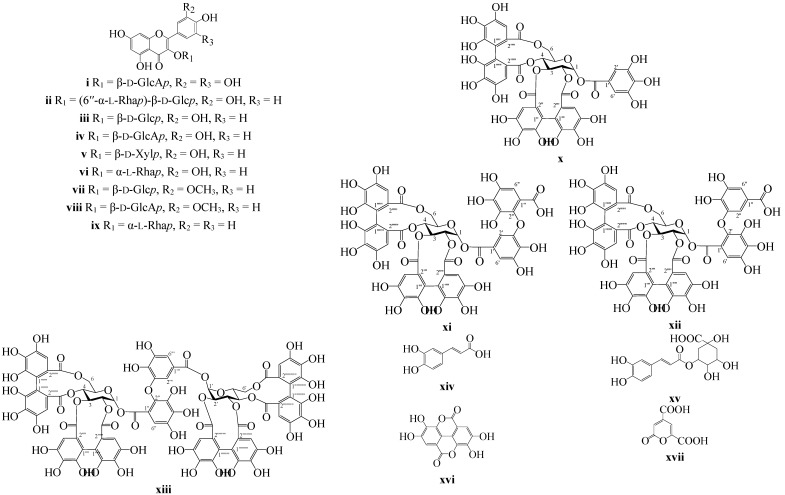
Chemical structures of compounds **i**–**xvii** isolated from *P. anserina* herb. β-d-Glcp—β-d-glucopyranose; β-d-GlcAp—β-d-glucuronopyranose; α-l-Rhap—α-l-rhamnopyranose; β-d-Xyl*p*—β-d-xylopyranose.

At the preliminary stage of the study we examined the phenolic profile of *P. anserina* collected in the Yakutian region of Siberia. The 60% MeOH-extract of *P. anserina* herb was partitioned with CHCl_3_, EtOAc, and *n*-BuOH to yield three fractions, which were separated by column chromatography (silica gel, Amberlite XAD7HP, polyamide, Sephadex LH-20 chromatography) and prep. HPLC, to give 17 compounds, with nine being flavonoids [myricetin-3-*O*-β-d-glucuronopyranoside (**i**), rutin (quercetin-3-*O*-rutinoside; (**ii**), isoquercitrin (quercetin-3-*O*-β-d-glucopyranoside; (**iii**), miquelianin (quercetin-3-*O*-β-d-glucuronopyranoside; (**iv**), reynoutrin (quercetin-3-*O*-β-d-xylopyranoside; (**v**), quercitrin (quercetin-3-*O*-α-l-rhamnopyranoside; (**vi**), isorhamnetin-3-*O*-β-d-glucopyranoside (**vii**), isorhamnetin-3-*O*-β-d-glucuronopyranoside (**viii**), kaempferol-3-*O*-α-l-rhamnopyranoside (**ix**)]. Additionally, four ellagitannins [potentillin (**x**), agrimonic acid A (**xi**), agrimonic acid B (**xii**), agrimoniin (**xiii**)], caffeic acid (**xiv**), 3-*O*-caffeoylquinic acid (**xv**), ellagic acid (**xvi**), 2-pyrone-4,6-dicarboxylic acid (**xvii**) were isolated and identified by comparison of their m.ps, optical rotation values, UV, ^1^H- and ^13^C-NMR spectroscopic, and MS data with those reported in the literature and reference samples (Figure 1).

**Table 1:** Known HPLC assays for analysis of *P. anserina* components.

Method [ref.]	Compound(s)	Column	Mobile Phases	Analysis Time, min
Quantitative techniques				
HPLC-UV [13]	2-Pyrone-4,6-dicarboxylic acid	ProSep C-18 (150 mm × 4 mm × 5 μm)	MeCN, 0.1% TFA	45
HPLC-UV [29]	Caffeic acid, ferulic acid, quercetin, rutin	Spherisorb^®^ ODS2 (250 mm × 4.6 mm × 5 μm)	1% AcOH, 6% AcOH, 5% AcOH in 30% MeCN	120
HPLC-UV [8]	Gallic acid, pedunculagin, catechin, ellagic acid, agrimoniin	Chromolith Performance RP-18e (100 mm × 4.6 mm)	0.2% HCOOH, 0.2% HCOOH in MeCN	32
HPLC-DAD [7]	Epigallocatechin, catechin, epicatechin, rutin, isoquercitrin, isorhamnetin-3-*O*-glucoside, kaempferol-3-*O*-glucoside, ellagic acid	Supelco LC RP 18 (250 mm × 4.6 μm × 5 μm)	10% MeCN, 55% MeCN	60
Qualitative techniques				
NanoLC-Orbitrap-MS [10]	Chlorogenic acid, myricetin-3-*O*-glucuronide, quercetin-3-*O*-sambubioside, myricitrin, isoquercitrin, miquelianin, reynoutrin, astragalin, rutin, isorhamnetin-3-*O*-glucuronide, linarin, nicotoflorin	Waters nano Acquity HSS T3 (100 mm × 100 μm × 1.8 μm)	0.1% HCOOH, 90% CH_3_CN	63
UHPLC-DAD-MS/MS [28]	Ellagic acid, agrimoniin	Kinetex C8 (100 mm × 2.1 mm × 1.7 μm)	0.1% HCOOH, 0.1% HCOOH in MeCN	35

All isolated flavonoids **i**–**ix**, phenylpropanoids **xiv**, **xv**, ellagic acid (**xvi**) and 2-pyrone-4,6-dicarboxylic acid (**xvii**) were detected in *P. anserina* herb in early studies of this species [6,10,12,13]. Three of the isolated ellagitannins (compounds **x**–**xii**) were detected in *P. anserina* the first time. Previously, compound **10** (pontetillin) was identified as a component of *P. kleiniana* [31]. Ellagitannin agrimoniin (**xiii**) isolated from the root of *Agrimonia japonica* (Miq.) Koidz. [32] was also detected in the herb and roots of some *Potentilla* species, including *P. kleiniana* [31], *P. anemonefolia*, *P. centrigrana* [32], *P. erecta* [33], *P. discolor* [34], and *P. recta* [35]. Recently, the presence of agrimoniin was detected in *P. anserina* herb [8]. Compounds **xi** (agrimonic acid A) and **xii** (agrimonic acid B) were not detected in *Potentilla* species before.

### 2.2. MC-RP-HPLC-UV Method Development and Validation

Method development was evaluated with a sample of ethanolic extract of the *P. anserina* herb. Optimum separation of the constituents of *P. anserina* herb was achieved by means of a rather complicated solvent gradient consisting of aqueous 0.2 М LiClO_4_ in 0.006 M HClO_4 _and acetonitrile as mobile phases. Replacement of acetonitrine by methanol resulted in the unsatisfactory resolution of adjacent peaks.

Application of isocratic conditions resulted in the elongation of analysis time and bed peak sharpness. Concerning the stationary phase, out of a number of different micro-HPLC columns tested (C-8, C-12, C-16, C-18), the best results were obtained with ProntoSIL-120-5-C18 AQ. It was also discovered that separation was better when column temperature was kept at 35 °C than lower (20°, 30 °C) or higher (40°, 45 °C).

The flow rate is an important parameter of a chromatographic process. The application of high flow rates resulted in the reduction of analysis time and more efficient obtainment of data. During the method development, we studied various flow rates from 50 μL·min^−1^ to 1 mL·min^−1^. As a result, a 0.6 mL·min^−1^ flow rate was chosen as the rate that gave the desired separation and rapid analysis time (5 min).

Preliminary experiments showed that only seven compounds isolated from *P. anserina* were in detectable amounts in extract, including caffeic acid, myricetin-3-*O*-glucuronide, agrimoniin, ellagic acid, miquelianin, isorhamnetin-3-*O*-glucuronide, and kaempferol-3-*O*-rhamnoside. Other identified components are characterized as trace components. According to the absorption maxima of the seven mentioned compounds on the UV-spectra, the monitoring wavelength was set at 270 nm.

**Figure 2:**
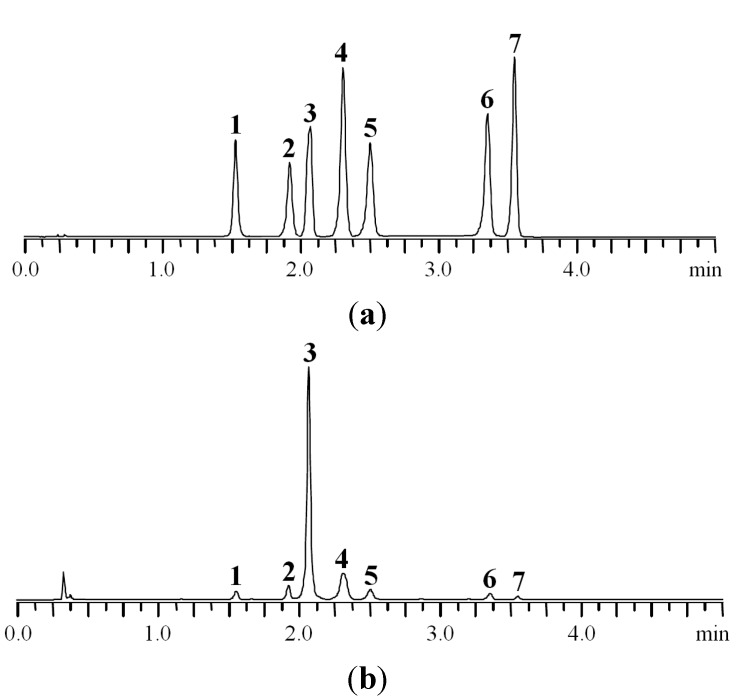
MC-RP-HPLC-UV chromatograms of standard mixture (**a**) and *P. anserina* extract (**b**) detected at 270 nm. **1**. caffeic acid; **2**. myricetin-3-*O*-glucuronide; **3**. agrimoniin; **4**. ellagic acid; **5**. miquelianin; **6**. isorhamnetin-3-*O*-glucuronide; **7**. kaempferol-3-*O*-rhamnoside.

Finally, the best separation conditions are the following: column ProntoSIL-120-5-C18 AQ (1 mm × 60 mm × 5 μm) eluted with 0.2 М LiClO_4_ in 0.006 M HClO_4_ (eluent A) and acetonitrile (eluent B) with a six-step gradient (0–1.25 min 11%–18% B, 1.25–2.25 min 18% B, 2.25–2.5 min 18%–20% B, 2.5–3.0 min 20%–25% B, 3.0–4.0 min 25% B, 4.0–5.0 min 25%–100% B) with flow rate 0.6 mL·min^−1^ and maintained at 35 °C. Representative chromatograms of standard solution and *P. anserina* extract are shown in Figure 2.

The proposed microcolumn RP-HPLC-UV method was validated in terms of linearity, precision, repeatability, recovery, and solution stability and robustness. The results of method validation are listed in Table 2 and Table 3.

**Table 2:** Linear regression data, LOD and LOQ of seven compounds from *P. anserina*.

Compound	Regression Equation	*r*^2^	*S_YX_*	Linear Range, μg/mL	LOD, μg/mL	LOQ, μg/mL
Caffeic acid	*Y* = 0.0203 × *X* − 0.0073	0.9999	0.28·10^−2^	2–1500	0.46	1.38
Myricetin-3-*O*-glucuronide	*Y* = 0.0194 × *X* − 0.0376	0.9999	1.14·10^−2^	10–2000	1.94	5.88
Agrimoniin	*Y* = 0.0307 × *X* − 0.0416	0.9999	1.22·10^−2^	5–2500	1.31	3.97
Ellagic acid	*Y* = 0.0647 × *X* − 0.0803	0.9998	1.36·10^−2^	2.5–1800	0.69	2.10
Miquelianin	*Y* = 0.0285 × *X* − 0.0363	0.9999	0.78·10^−2^	5–1500	0.90	2.74
Isorhamnetin-3-*O*-glucuronide	*Y* = 0.0328 × *X* − 0.0190	0.9999	0.88·10^−2^	5–1500	0.89	2.68
Kaempferol-3-*O*-rhamnoside	*Y* = 0.0402 × *X* − 0.0119	0.9999	0.26·10^−2^	2–1000	0.21	0.65

**Table 3:** Precision and repeatability data of seven compounds from *P. anserina*.

Compound	Repeatability, %	Variability, %	Intra-Day Precision, %	Inter-Day Precision, %
Caffeic acid	1.03	2.04	1.35	1.57
Myricetin-3-*O*-glucuronide	1.49	1.57	1.93	2.14
Agrimoniin	2.02	1.93	2.20	2.57
Ellagic acid	2.17	2.63	2.34	2.72
Miquelianin	1.56	1.11	1.53	2.11
Isorhamnetin-3-*O*-glucuronide	0.92	0.71	1.69	2.09
Kaempferol-3-*O*-rhamnoside	0.67	0.52	1.43	1.87

A linear relationship was obtained between a response (peak area) and the amount of phenolic compounds in the ranges 2–1500, 10–2000, 5–3000, 2.5–1800, 5–1500, 5–1500, and 2–1000 μg/mL for caffeic acid, myricetin-3-*O*-glucuronide, agrimoniin, ellagic acid, miquelianin, isorhamnetin-3-*O*-glucuronide, and kaempferol-3-*O*-rhamnoside, respectively. The correlation coefficients were 0.9999 for caffeic acid, myricetin-3-*O*-glucuronide, agrimoniin, miquelianin, isorhamnetin-3-*O*-glucuronide, and kaempferol-3-*O*-rhamnoside, and 0.9998 for ellagic acid. Limits of detection (LOD) of caffeic acid, myricetin-3-*O*-glucuronide, agrimoniin, ellagic acid, miquelianin, isorhamnetin-3-*O*-glucuronide, and kaempferol-3-*O*-rhamnoside were 0.46, 1.94, 1.31, 0.69, 0.90, and 0.89 μg/mL, respectively.

Intra- and inter-day variations, which are presented in terms of percent relative standard deviation (%RSD) of the analyte’s peak area and variability were chosen to determine the precision of the developed method. For intra-day variability test, the calibration sample solutions were analysed for five replicates within one day, while the solutions were examined in duplicates for three consecutive days for inter-day variability tests. Values of intra- and inter-day precision were 1.35–2.34 and 1.57%–2.72%, respectively. The repeatability of the method was 0.67%–2.17%, while the variability was 0.52%–2.63%.

The recovery was determined by the standard addition method. Seven phenolic compounds were spiked into the samples, and then extracted, processed, and quantified in accordance with the established procedures. Each sample was analyzed five times to determine the mean content (μg/mL). The RSD value was calculated as a measurement of method repeatability. The results are presented in Table 4.

**Table 4:** Recoveries of seven compounds in *P. anserina*.

Compound	Original Content, μg/mL	Spiked, μg/mL	Detected, μg/mL	Recovery, %	RSD, %
Caffeic acid	106.55	60	167.42	100.52	1.16
		90	194.16	98.78	1.31
		120	221.02	97.56	1.69
Myricetin-3-*O*-glucuronide	316.56	160	484.37	101.64	2.02
		240	553.28	99.41	1.63
		320	623.90	98.01	1.84
Agrimoniin	1026.34	500	1542.37	101.05	1.63
		750	1806.22	101.68	1.92
		1000	2074.58	102.38	2.14
Ellagic acid	216.03	110	320.62	98.34	1.12
		165	377.75	99.14	1.67
		220	441.43	101.24	1.90
Miquelianin	165.34	80	239.26	97.52	1.31
		120	280.03	98.14	2.10
		160	328.33	100.92	2.16
Isorhamnetin-3-*O*-glucuronide	69.77	40	107.86	98.26	1.09
		60	126.07	97.15	1.52
		80	148.53	99.17	1.16
Kaempferol-3-*O*-rhamnoside	20.94	10	31.44	101.62	1.34
		15	35.72	99.39	0.92
		20	41.73	101.93	1.27

The stability of standard and sample solutions was determined by monitoring the peak area of standard mixture solutions and retention time with sample solutions over a period of one day. The results showed that the peak area and retention time of each analyte were almost unchanged. The concentration of substances after each storage period was related to the initial analyte concentrations of freshly prepared samples. Samples were stable within the acceptable limits of accuracy and precision at least in 24 h.

The results of method validation of qualitative and quantitative analysis showed that the microcolumn RP-HPLC-UV method was precise, recovery and accurate enough for the simultaneous quantitative and qualitative evaluation of seven phenolic compounds in *P. anserina* herb.

### 2.3. Optimisation of Extraction Procedure of the Main Phenolic Compounds from the P. anserina Herb

Various solvents, extraction methods, temperatures, and times were evaluated to obtain the best extraction efficiency. The results of the determination of the best extraction type revealed that sonification was better than refluxing and stirring, so the subsequent experiments were carried out with sonification.

According to the literature data there is no “common” solvent for extraction of *P. anserina* phenolic compounds; separate authors used 70% acetone [13], 30%–100% methanol [7,8], 70% ethanol [10], and water [28]. For this reason, different media, including conventional solvents as water and alcohols, as well as uncommon solvents like ketones, ethers, and DMSO, were screened (Table 5). The results achieved for the aqueous extract showed the highest content of caffeic acid (152.68 μg/mL); the concentration of flavonoids, agrimoniin, and ellagic acid is much lower than that of the methanol extract. Ethanol extract is characterized by the intermediate values of the concentrations of all compounds except ellagic acid, the concentration of which is lower than in water extraction (7.89 μg/mL). The values of the total content of phenolics were 639.27, 1123.04, and 585.60 μg/mL in water, methanol, and ethanol extract, respectively. Application of the *n*-propanol and *iso*-propanol did not allow for the obtaining of the extacts with higher content; total identified compounds content in *n*-propanol and *iso*-propanol extracts were 122.62 and 26.41 μg/mL, respectively. The extraction power of other solvents decreased sequentially in line: *n*-propanol >> *iso*-propanol ≈ acetone > ethylacetate > methylethylketone > acetonitrile. The latter solvent is characterized by traced ability to extraction of *P.* *anserina* phenolics.

The use of polar aprotic solvent DMSO allows for the preparation of extract with the highest total phenolic content, 1806.23 μg/mL. However, the content of caffeic acid (135.61 μg/mL) was lower than in water extract. In a further step, we analyzed a quantitative compositions of extracts prepared on water-containing solvent at a 50% level of water content (just water miscible solvents were used). As a result, a significant increase of phenolic content was observed for all solvents. The extraction power of 50% solvent presented in the following manner: acetone > DMSO > acetonitrile > *n*-propanol > ethanol > *iso*-propanol > methanol. Maximal content of all analyzing compounds was detected in acetone containing media that were determined as perspective solvents and examined in further experiments.

To obtain more specific information about optimal acetone concentrations, the various dilutions of acetone were used as solvents for extraction of *P.* *anserina* phenolics. When the concentration of acetone increased from 0% to 60%, the yield of phenols increased significantly, reaching a maximum value, 2949.76 μg/mL, at 60% acetone. Then, an obvious decrease of all compounds content with further increases of the acetone concentration from 60% to 90% was observed. Thus, 60% acetone is an appropriate solvent for the extraction of *P.* *anserina* phenolics.

**Table 5:** Content of seven compounds in *P.* *anserina* extracts, μg/mL *.

Solvent	Compound **							
	1	2	3	4	5	6	7	Total
100% solvent								
Water	152.68	71.36	281.19	80.07	23.10	28.59	2.28	639.27
Methanol	44.36	92.54	760.95	106.97	61.39	49.06	7.77	1123.04
Ethanol	133.65	54.00	304.12	7.89	35.01	45.30	5.63	585.60
*n*-Propanol	6.11	65.05	24.43	11.91	7.09	8.03	tr.	122.62
*iso*-Propanol	tr.	8.26	2.47	6.72	3.64	5.32	tr.	26.41
Acetone	tr.	2.50	11.82	6.39	tr.	tr.	4.11	24.82
Methylethylketone	tr.	tr.	5.63	tr.	tr.	2.31	tr.	7.94
Acetonitrile	tr.	n.d.	tr.	tr.	tr.	tr.	n.d.	tr.
Ethylacetate	tr.	6.88	4.11	tr.	tr.	tr.	tr.	10.99
DMSO	135.61	243.29	1004.63	189.40	140.40	74.78	18.12	1806.23
50% solvent								
Methanol	150.87	221.16	901.55	152.78	135.84	67.45	21.56	1651.21
Ethanol	157.35	230.26	1040.53	183.61	135.12	67.00	23.76	1837.63
*n*-Propanol	123.16	260.76	1153.46	204.15	160.15	79.22	25.34	2006.24
*iso*-Propanol	185.43	216.12	927.21	194.99	144.03	72.87	25.14	1765.79
Acetone	189.26	264.17	1713.24	210.76	161.31	79.82	25.52	2644.08
Acetonitrile	142.40	247.72	1315.63	191.51	139.75	74.78	21.50	2133.29
DMSO	180.70	247.94	1611.49	187.19	143.78	62.52	23.97	2457.59
Acetone. Various concentrations								
20%	180.33	218.78	887.82	148.09	129.54	60.93	21.11	1646.60
30%	183.55	224.61	1127.97	173.80	131.63	61.14	22.39	1925.09
40%	185.26	237.73	1578.04	202.97	154.53	74.04	25.61	2458.18
60%	190.49	296.93	1953.69	229.07	169.21	81.97	28.40	2949.76
70%	114.05	252.02	1824.03	212.02	123.24	48.89	25.09	2599.34
80%	93.42	149.39	1821.94	180.95	86.12	28.69	19.44	2379.95
90%	21.16	41.16	126.11	56.09	10.57	12.10	5.63	272.82

* Extraction conditions: liquid-to-solid ratio 1:25, extraction temperature 40 °C, extraction time 30 min; ** 1. caffeic acid; 2. myricetin-3-*O*-glucuronide; 3. agrimoniin; 4. ellagic acid; 5. miquelianin; 6. isorhamnetin-3-*O*-glucuronide; 7. kaempferol-3-*O*-rhamnoside.

**Figure 3:**
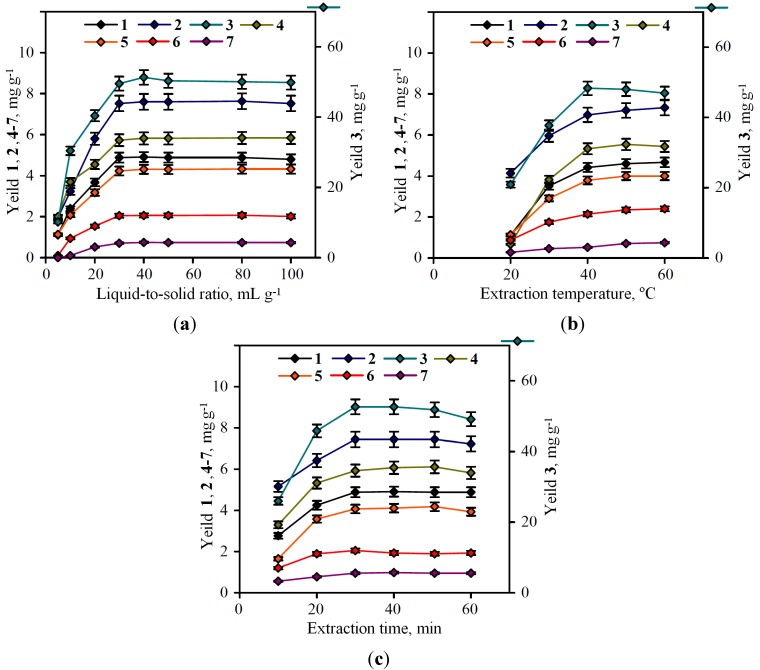
Effects of extraction parameters on the phenolic compounds yield: (**a**) effect of liquid-to-solid ratio; (**b**) effect of extraction temperature; (**c**) effect of extraction time. 1. caffeic acid; 2. myricetin-3-*O*-glucuronide; 3. agrimoniin; 4. ellagic acid; 5. miquelianin; 6. isorhamnetin-3-*O*-glucuronide; 7. kaempferol-3-*O*-rhamnoside.

To determine the effect of the liquid-to-solid ratio on extraction yield, experiments were carried out at a ratio ranging between 1:5 and 1:100. As shown in Figure 3a, the extraction yield initially increased when the ratio increased from 1:5 to 1:40, and then remained fairly constant for all compounds except agrimoniin. The further increasing of the liquid-to-solid ratio resulted in the decreasing of agrimoniin content (from 51.31 mg·g^−1^ at 1:40 to 49.81 mg·g^−1^ at 1:100). Therefore, the liquid-to-solid ratio of 1:40 was chosen for further optimization studies.

The influence of extraction temperature on the efficacy of extraction was investigated. Samples of *P. anserina* were sonificated with 60% acetone in liquid-to-solid ratio 1:40 at 20–60 °C. The limitation of temperature interval by 60 °C point induced by the boiling point of 60% acetone which is approximately 62–65 °C. The results obtained were variable for different compounds (Figure 3b).

The maximal agrimoniin content was detected in 40 °C-extract, 50.32 ± 1.18 mg·g^−1^; then we observed the reduction of agrimoniin content up to 46.74 ± 1.07 mg·g^−1^ at 60 °C. For some flavonoids (myricetin-3-*O*-glucuronide, isorhamnetin-3-*O*-glucuronide, kaempferol-3-*O*-rhamnoside) in a 40–60 °C interval, the increasing of extraction temperature up to 60 °C resulted in the rising of the concentration (for myricetin-3-*O*-glucuronide from 7.02 mg·g^−1^ to 7.23 mg·g^−1^; for isorhamnetin-3-*O*-glucuronide from 2.14 mg·g^−1^ to 2.21 mg·g^−1^; for kaempferol-3-*O*-rhamnoside from 0.52 mg·g^−1^ to 0.54 mg·g^−1^). The reason for these changes is the greater stability of flavonoids to the influence of temperature factor as opposed to ellagitannins. The differences of concentration in 40 °C- and 60 °C-extracts for the mentioned compounds are −3.58 mg·g^−1^, +0.21 mg·g^−1^, +0.07 mg·g^−1^, and +0.02 mg·g^−1^, respectively, for agrimoniin, myricetin-3-*O*-glucuronide, isorhamnetin-3-*O*-glucuronide, and kaempferol-3-*O*-rhamnoside. In consideration of the more expressed value of concentration lost for agrimoniin, we decided to choose 40 °C as an optimal temperature point.

In order to obtain the maximum yield of phenolic compounds from *P. anserina*, sonification was performed with 60% acetone in the liquid-to-solid ratio 1:40 at 40 °C and at six extraction times from 10 to 60 min (Figure 3c). It was revealed that the period 30 min of extraction time favours the phenolic compounds’ production. Further increasing the extraction time did not show a significant increase in the flavonoid and phenolic acid content. Moreover, the decreasing of agrimoniin content was observed after 30 min of extraction. Therefore, 30 min was chosen as the extraction time in subsequent experiments.

### 2.4. Phenolic Content in P. anserina Herb and Some Preparations

The developed method was successfully applied to the analysis of 12 batches of the *P. anserina* herb collected from three Siberian regions (Yakutia, Buryatia, Irkutsk) and two commercial samples. The desired components in *P. anserina* herb were identified by comparing both the retention times and UV spectra of phenolic compounds with those of the reference standards. To check the peak purity, the eluates were monitored at 200–400 nm in upslope, apex, and downslope of each peak, and the resulting three spectra were normalised and superimposed. A peak was considered pure when there was an exact coincidence among three spectra [36]. The results showed the presence of seven required compounds in all analyzed batches of *P. anserina* herb (Table 6). The peak’s purity was satisfactory for quantitative analysis of samples.

**Table 6:** Content of seven compounds in *P. anserina* herb, mg·g^−1^.

Batch No	Compound *							
	1	2	3	4	5	6	7	Total
PA-Y-01(2013)	2.14 ± 0.05	5.20 ± 0.09	53.12 ± 1.12	4.83 ± 0.12	3.15 ± 0.07	1.18 ± 0.03	0.92 ± 0.02	70.54
PA-Y-02(2013)	3.18 ± 0.07	6.19 ± 0.11	57.29 ± 1.26	3.02 ± 0.07	4.18 ± 0.09	1.37 ± 0.03	1.52 ± 0.04	76.75
PA-Y-03(2014)	4.12 ± 0.09	7.93 ± 0.15	51.14 ± 1.02	3.12 ± 0.06	3.07 ± 0.07	2.69 ± 0.06	1.93 ± 0.04	74.00
PA-Y-04(2014)	4.76 ± 0.10	7.42 ± 0.14	48.83 ± 1.07	5.77 ± 0.14	4.23 ± 0.09	2.05 ± 0.05	0.71 ± 0.02	73.77
PA-B-05(2012)	4.97 ± 0.11	8.63 ± 0.16	37.19 ± 0.81	3.21 ± 0.08	5.93 ± 0.12	3.37 ± 0.07	2.41 ± 0.06	65.71
PA-B-06(2013)	5.39 ± 0.11	9.10 ± 0.18	30.06 ± 0.72	2.93 ± 0.07	6.29 ± 0.15	3.30 ± 0.07	1.98 ± 0.04	59.05
PA-B-07(2014)	5.64 ± 0.12	8.54 ± 0.17	40.14 ± 0.84	2.44 ± 0.06	6.54 ± 0.16	2.97 ± 0.06	2.52 ± 0.05	68.79
PA-B-08(2014)	5.97 ± 0.14	8.29 ± 0.16	22.17 ± 0.42	1.92 ± 0.04	6.97 ± 0.19	3.72 ± 0.08	2.67 ± 0.05	51.71
PA-I-09(2012)	6.18 ± 0.12	8.90 ± 0.17	20.69 ± 0.46	1.84 ± 0.04	6.38 ± 0.14	4.69 ± 0.09	3.74 ± 0.08	52.42
PA-I-10(2013)	5.32 ± 0.11	9.21 ± 0.17	18.33 ± 0.38	1.24 ± 0.03	7.52 ± 0.18	5.27 ± 0.12	3.11 ± 0.07	50.00
PA-I-11(2014)	5.18 ± 0.10	10.36 ± 0.21	22.94 ± 0.55	0.97 ± 0.02	8.12 ± 0.19	5.22 ± 0.12	3.62 ± 0.08	56.41
PA-I-12(2014)	6.04 ± 0.14	6.52 ± 0.12	25.11 ± 0.59	1.25 ± 0.03	8.04 ± 0.17	4.37 ± 0.10	3.68 ± 0.08	55.01
PA-C-13(2014)	3.18 ± 0.05	7.52 ± 0.17	32.57 ± 0.68	2.61 ± 0.06	3.36 ± 0.07	2.10 ± 0.05	1.37 ± 0.03	52.71
PA-C-14(2014)	5.22 ± 0.10	4.18 ± 0.08	41.17 ± 0.82	3.79 ± 0.10	3.14 ± 0.05	1.94 ± 0.04	0.62 ± 0.01	60.06

* 1. caffeic acid; 2. myricetin-3-*O*-glucuronide; 3. agrimoniin; 4. ellagic acid; 5. miquelianin; 6. isorhamnetin-3-*O*-glucuronide; 7. kaempferol-3-*O*-rhamnoside.

The data obtained demonstrated the fact of agrimoniin domination in all samples of *P. anserina* herb in a wide range of concentrations, 18.33–57.29 mg·g^−1^. The variation of flavonoid and phenolic acid content was as follows: caffeic acid 2.14–6.18 mg·g^−1^, myricetin-3-*O*-glucuronide 4.18–10.36 mg·g^−1^, ellagic acid 0.97–5.77 mg·g^−1^, miquelianin 3.07–8.12 mg·g^−1^, isorhamnetin-3-*O*-glucuronide 1.18–5.27 mg·g^−1^, and kaempferol-3-*O*-rhamnoside 0.62–3.74 mg·g^−1^. The minimal value of the total phenolic content was observed in the Irkutsk sample PA-I-10(2013), 50.00 mg·g^−1^, and the maximal content was marked in the Yakutia sample PA-Y-02(2013), 76.75 mg·g^−1^.

**Figure 4:**
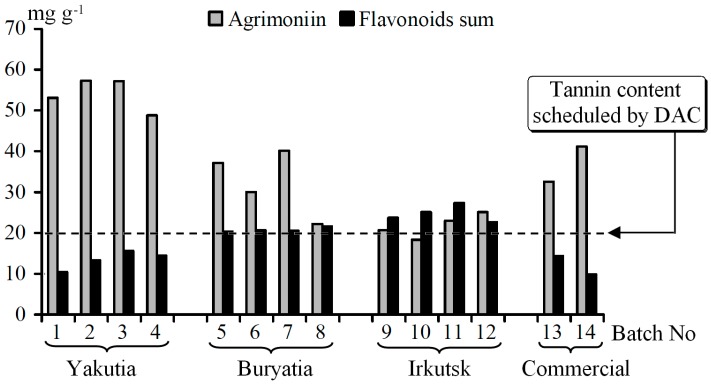
Content of agrimoniin and flavonoids sum in *P. anserina* herb batches.

It should be noted that the samples of *P. anserina* collected in three Siberia regions were characterized by different abilities to accumulate the phenolic compounds (Figure 4). The Yakutian populations contained high concentrations of agrimoniin (48.83–57.29 mg·g^−1^) and low concentrations of flavonoids (10.45–15.62 mg·g^−1^). An opposite character of accumulation was observed in the Irkutsk populations, *viz.* low agrimoniin content (18.33–25.11 mg·g^−1^) and high flavonoid content (22.61–27.32 mg·g^−1^). The plant populations growing in Buryatia occupied an intermediate position (agrimoniin 22.17–40.14 mg·g^−1^; flavonoids 20.34–21.65 mg·g^−1^). Early data about the chemical profile of the *P. anserina* herb growing in different regions indicate a high content of tannins in Polish populations (1.75%–2.26%) [8] and a low content of flavonoids (0.48%–0.60%), respectively [7]. *P. anserina* of German origin were characterized as a high-flavonoid containing population (>1%), while in the Ukrainian samples the content of flavonoids were on the lowest level (0.35%) [37]. According to the German Drug Codex Supplement to Pharmacopoeia (DAC), the tannin content in *P. anserina* herb should not be less than 2% [38]. Therefore, Polish and Siberian populations (except some Irkutsk batches) of *P. anserina* may be regarded as satisfactory.

Chromatographic analysis of flowers, leaves, and stems of *P. anserina* showed that none of the investigated plant parts contained additional phenolic constituents; only variations in the content of the seven analyzed compounds were observed (Table 7). The highest content of agrimoniin was detected in leaves (61.27 mg·g^−1^), followed by stems (25.03 mg·g^−1^) and flowers (21.98 mg·g^−1^). The distribution of phenolic acids and flavonoids was different; the maximal concentrations were in flowers and the minimal ones in stems. The tannin:flavonoid ratio in the flowers, leaves, and stems was ~1:1, 4.3:1, and 3.6:1, respectively.

**Table 7:** Content of seven compounds in *P. anserina* organs, mg·g^−1^ *.

Compound	Flowers	Leaves	Stems
Caffeic acid	8.89 ± 0.19	7.63 ± 0.15	0.82 ± 0.02
Myricetin-3-*O*-glucuronide	10.99 ± 0.21	7.72 ± 0.14	3.50 ± 0.07
Agrimoniin	21.98 ± 0.39	61.27 ± 1.16	25.03 ± 0.47
Ellagic acid	5.93 ± 0.15	5.62 ± 0.14	4.50 ± 0.10
Miquelianin	6.51 ± 0.12	3.27 ± 0.06	2.06 ± 0.04
Isorhamnetin-3-*O*-glucuronide	2.75 ± 0.05	2.67 ± 0.05	1.17 ± 0.02
Kaempferol-3-*O*-rhamnoside	1.56 ± 0.04	0.45 ± 0.01	0.31 ± 0.01
Total content	58.61	88.63	37.39

* sample PA-Y-02(2013).

The previously mentioned differences in phenolic compounds content in *P. anserina* collected in various countries might result from the environmental effects, and the extraction conditions, as well as different development stadium of the investigated plants or the different ratios of flowers, leaves, and stems in the analyzed samples. Given the widespread use of preparations from *P. anserina* in therapeutic practice, we studied the quantitative content of phenolic compounds in five medical forms, including ethanol containing forms (liquid extract and tincture), decoction, and infusion as frequently applied home-made forms and dry extract used for tablet and pill manufacturing. The qualitative composition of the analyzed preparations from *P. anserina* was similar to those of native plant material. This fact indicates the safety of the componential profile of the analyzed preparations within the manufacturing process.

The most enriched liquid formulation was liquid extract characterized by 5.86 mg/mL of total phenolic content (Table 8). Tincture, decoction, and infusion are dosage forms prepared by low-extensive technology that affects the quality of the resulting product. The total content of agrimoniin in infusion as the most depleted form was 0.11 mg/mL, which was almost 23 times smaller than that on the liquid extract (2.34 mg/mL). The differences in total flavonoid content between infusion and liquid extract were less pronounced: 0.66 mg/mL and 2.05 mg/mL, respectively. Tincture and decoction are the medical preparations with intermediate quantitative parameters.

**Table 8:** Content of seven compounds in *P. anserina* preparations.

Compound	Liquid Extract, μg/mL	Tincture, μg/mL	Decoction, μg/mL	Infusion, μg/mL	Dry Extract, mg·g^−1^
Caffeic acid	767.85 ± 16.12	442.48 ± 8.84	365.49 ± 7.13	287.82 ± 6.04	13.58 ± 0.29
Myricetin-3-*O*-glucuronide	955.30 ± 18.15	614.49 ± 13.52	443.14 ± 7.09	330.08 ± 6.93	24.22 ± 0.39
Agrimoniin	2388.68 ± 54.93	1846.76 ± 38.78	180.24 ± 4.59	105.70 ± 2.53	84.12 ± 1.76
Ellagic acid	649.28 ± 17.53	381.60 ± 9.15	268.85 ± 6.45	186.67 ± 3.73	17.56 ± 0.40
Miquelianin	620.76 ± 14.27	329.65 ± 6.92	272.11 ± 5.98	206.41 ± 3.50	13.25 ± 0.32
Isorhamnetin-3-*O*-glucuronide	336.43 ± 6.39	168.90 ± 4.39	116.32 ± 1.98	86.14 ± 1.98	6.88 ± 0.16
Kaempferol-3-*O*-rhamnoside	140.93 ± 3.38	63.83 ± 1.15	46.17 ± 1.24	33.46 ± 0.70	2.18 ± 0.06
Total content	5859.23	3847.71	1692.32	1236.28	161.79

The different relative content of agrimoniin on the total content of identified compounds was observed in water- and ethanol-extracted preparations. This parameter was 10.6% and 8.6% in decoction and infusion, respectively,* vs.* 40.8% and 48.0% in liquid extract and tincture, respectively. The possible reason for these differences is the limited effectiveness of water as an extractant for isolation of ellagitannins from a plant matrix. Furthermore, we allow for the fact of the thermal destruction of ellagitannins within the preparation of decoction and infusion due to high temperature process.

The dry extract of *P. anserina* herb was the most enriched formulation containing 16.18% of total phenolics, including 8.41% of agrimoniin, 4.65% of flavonoids, 1.36% of caffeic acid, and 1.76% of ellagic acid. Actual information on the acceptable uptake of various formulations of *P. anserina* [39] allowed us to calculate values of daily consumption of the main groups of active compounds. The results presented in Table 9 show that despite on archaic character of infusion and decoction as medical formulations, their application maximizes uptake values of tannins and flavonoids in 40–60 times greater than after using liquid extract or tincture. Interestingly, despite the fact of the high content of phenolics in dry extract, the daily uptake of flavonoids and total phenols is lower than in the case of infusions or decoctions.

**Table 9:** Daily uptake of phenolic compounds after application of *P. anserina* preparations.

Preparation	Recommended Daily Uptake of Preparation	Daily Uptake of Compounds, mg·g^−1^·day^−1^		
		Tannins	Flavonoids	Total Phenols
Liquid extract	1–2 mL	2.3–4.8	2.1–4.1	5.9–11.7
Tincture	2–3 mL	3.7–5.5	2.4–3.5	7.7–11.5
Decoction	100–150 mL	18.0–27.0	87.7–131.5	169.2–254.9
Infusion	100–150 mL	10.6–15.9	65.6–98.4	123.6–185.4
Dry extract	0.5–1 g	42.1–84.1	23.3–46.5	80.9–161.8

These data demonstrate the possibility of adequate substitution of liquid extracts or tinctures by infusions or decoctions at inability of application of the ethanol-containing formulations (children’s therapy, allergy to ethanol,* et al.*).

## 3. Experimental Section

### 3.1. General

Elemental (C/H/O) composition was determined using MAT 8200 spectrometer (Thermo Finnigan, Waltham, MA, USA). UV spectra were recorded using a SF-2000 spectrophotometer (OKB Specter, St. Petersburg, Russia). Optical rotations were measured on 341 Series polarimeter (Perkin Elmer, Waltham, MA, USA). MS spectra were registered on a LCQ mass spectrometer (Thermo Finnigan). NMR spectra were recorded on a VXR 500S spectrometer (Varian, Palo Alto, CA, USA). Column chromatography was performed over silica gel 60 (230–400 mesh, Merck, Whitehouse Station, NJ, USA), Sephadex LH-20 (25–100 μm, Pharmacia, Uppsala, Sweden), polyamide Woelm (Waters Associates, Inc., Framingham, MA, USA), and Amberlite XAD1180N (Sigma-Aldrich, St. Louis, MO, USA). Finally, pTLC was performed on Sorbfil-A silica gel TLC plates (layer thickness 2 mm; Imid Ltd., Krasnodar, Russia). All chemicals were analytical-grade. Reference compounds with purity greater than 96% were used. This included commercially available compounds: caffeic acid, myricetin-3-*O*-glucuronide, ellagic acid, miquelianin, isorhamnetin-3-*O*-glucuronide, kaempferol-3-*O*-rhamnoside from Sigma-Aldrich (St. Louis, MO, USA); agrimoniin from Chelwill Asia Co., Ltd. (Beijing, China).

### 3.2. Plant Material

The samples of *Potentilla anserina* L. herb were collected in the flowering period in three Siberian regions of Russian Federation: *Yakutia Republic*—Churapcha village [Churapcha District; 25.VII.2013, 62°0278′34″ N, 132°48′90″ E, voucher specimen No Lm/h-905/0515; batch No PA-Y-01(2013)]; Amga village [Amga District; 26.VII.2013, 60°8992′97″ N, 131°98′52″ E, voucher specimen No Lm/h-905/0615; batch No PA-Y-02(2013)]; Lensk city [Lensk District; 31.VII.2014, 60°7355′60″ N, 114°92′17″ E, voucher specimen No Lm/h-905/0715; batch No PA-Y-03(2014)]; Aldan city [Aldan District; 02.VIII.2014, 58°6407′46″ N, 125°45′22″ E, voucher specimen No Lm/h-905/0815; batch No PA-Y-04(2014)]; *Buryatia Republic*—Ivolginsk village [Ivolginsk District; 25.VII.2012, 51°7917′24″ N, 107°23′71″ E, voucher specimen No Lm/h-122/0315; batch No PA-B-05(2012)]; Kurumkan village [Kurumkan District; 29.VII.2013, 54°3051′79″ N, 110°30′72″ E, voucher specimen No Lm/h-122/0415; batch No PA-B-06(2013)]; Kabansk village [Kabansk District; 29.VII.2014, 52°0583′54″ N, 106°60′33″ E, voucher specimen No Lm/h-122/0515; batch No PA-B-07(2014)]; Horinsk village [Horinsk District; 30.VII.2014, 52°1550′25″ N, 109°69′87″ E, voucher specimen No Lm/h-122/0615; batch No PA-B-08(2014)]; *Irkutsk oblast*’—Kaltuk village [Bratsk District; 27.VII.2012, 55°6800′36″ N, 101°74′08″ E, voucher specimen No Lm/h-115/0315; batch No PA-I-09(2012)]; Odinsk village [Angarsk District; 28.VII.2013, 52°4587′98″ N, 103°72′91″ E, voucher specimen No Lm/h-115/0415; batch No PA-I-10(2013)]; Tulun village [Tulun District; 30.VII.2014, 54°5642′43″ N, 100°53′50″ E, voucher specimen No Lm/h-115/0515; batch No PA-I-11(2014)]; Kuitun village [Kuitun District; 31.VII.2014, 54°3467′96″ N, 101°55′07″ E, voucher specimen No Lm/h-115/0615; batch No PA-I-12(2014)]. Commercial samples of *P. anserina* herb and preparations were purchased in Company Orokto [Irkutsk, Russua; herb, batch No PA-C-13(2014)], Public Corporation Krasnogorskleksredstva [Moscow, Russia; herb, batch No PA-C-14(2014)], Closed Corporation Vifitekh (Moscow, Russia; tincture) and Limited Liability Company Arura (Ulan-Ude, Russia; liquid extract, dry extract).

### 3.3. Extraction and Isolation

The Yakutian sample [PA-Y-02(2013)] of *P. anserina* herb (1.64 kg) were air-dried, ground, and extracted with 20 L of 70% acetone [40] at 40 °C two times (90 min each) on water bath with continuous stirring and the extracts were concentrated under reduced pressure to yield 541 g of crude extract. The crude extract was resuspended in water (1:5, *v*/*v*) and successively partitioned with hexane and EtOAc. The organic layers were dried *in vacuo* to yield 21.1 and 164.6 g of hexane and EtOAc fraction residues respectively. The EtOAc fraction (110 g) was chromatographed over Sephadex LH-20 (8 cm × 90 cm), eluting with 95% ethanol, 80% acetone and 50% acetone to obtain 3 fractions (fr. 1, 14 g; fr. 2, 63 g; fr. 3, 27 g). Fraction 2 (60 g) was re-chromatographed on a Sephadex LH-20 (5 cm × 110 cm), eluting with acetone-water (100:0→0:100) to obtain 11 fractions (frs. 2/1–2/11). Fr. 2/3 was chromatographed on pHPLC (Summit HPLC-system with UV-Vis detector (Dionex, Sunnyvale, CA, USA), column LiChrosorb RP-18 (10 mm × 250 mm, 7 μm, Merck), T 35 °C, flow rate 2 mL/min; solvent, linear gradient of 5%–80% of MeCN in H_2_O for 90 min; detector at 270 nm) to give 20 frs. (frs. 2/3-1–2/3-20). Fr. 2/3-11 (t*_R_* 31–34 min) was re-chromatographed in the same conditions to give agrimoniin (**xiii**; 3.14 g) [31]. Fr. 2/5 was chromatographed on pHPLC to give 20 frs. (frs. 2/5-1–2/5-20). Frs. 2/5-6–2/5-7 (t*_R_* 24–29 min) were combined and re-chromatographed (pHPLC) to give agrimonic acid B (**xii**; 102 mg) [31,40]. Frs. 2/5-8–2/5-9 (t*_R_* 29–31 min) were combined and re-chromatographed (pHPLC) to give agrimonic acid A (**xi**; 164 mg) [31,40]. Fr. 2/8 was chromatographed on pHPLC to give 20 frs. (frs. 2/8-1–2/8-20). Frs. 2/8-9 (t*_R_* 35–39 min) was re-chromatographed (pHPLC) to give potentillin (**x**; 54 mg) [31]. Fr. 3 (25 g) was subjected on a polyamide column (500 g), eluting with H_2_O (12 L), 40% EtOH (21 L) and 90% EtOH (7 L). These elutes were brought dried *in vacuo* to yield 1.4, 14.6 and 7.3 g of H_2_O (3/1), 40% EtOH (3/2) and 90% EtOH fraction (3/3) residue respectively. Fr. 3/1 (1.2 g) was subjected on an Amberlite XAD1180N column (100 g) preconditioned with 90% ethanol and water, eluting with H_2_O (1 L), 40% EtOH (1.5 L) and 90% EtOH (1 L). These elutes were brought dried *in vacuo* to yield 655, 308 and 101 mg of H_2_O (3/1-1), 40% EtOH (3/1-2) and 90% EtOH fraction (3/1-3) residue respectively. Fr. 3/1-2 (300 mg) was chromatographed on pHPLC (Summit HPLC-system with UV-Vis detector (Dionex, Sunnyvale, CA, USA), column LiChrosorb RP-18 (10 mm × 250 mm, 7 μm, Merck), T 35 °C, flow rate 2 mL/min; solvent, linear gradient of 5%–10% of MeCN in H_2_O for 50 min; detector at 316 nm) to give 10 frs. [frs. 3/1-2(1)–3/1-2(10)]. Fr. 3/1-2(3)–3/1-2(5) (t*_R_* 15–22 min) were combined and re-chromatographed in the same conditions to give 2-pyrone-4,6-dicarboxylic acid (**xvii**; 52 mg) [13]. Fr. 3/2 (14 g) was chromatographed over silica column (3 cm × 100 cm), eluting with CHCl_3_-MeOH (100:0→0:100) to obtain 11 fractions (frs. 3/2-1–3/2-11). Fr. 3/2-3 was crystallized from MeOH to give reynoutrin (quercetin-3-*O*-β-d-xylopyranoside; **v**; 11 mg) [41]. Frs. 3/2-4–3/2-5 were combined and chromatographed over Sephadex LH-20 (2 cm × 60 cm), eluting with ethanol-water (90:10→10:90) to give miquelianin (quercetin-3-*O*-β-d-glucuronopyranoside; **iv**; 129 mg) [42], ellagic acid (**xvi**; 63 mg) [43] and isoquercitrin (quercetin-3-*O*-β-d-glucopyranoside; **iii**; 27 mg) [44]. Fr. 3/2-6 was chromatographed over Sephadex LH-20 (2 × 60 cm), eluting with ethanol-water (90:10→10:90) to give rutin (quercetin-3-*O*-rutinoside; **ii**; 29 mg) [44] and caffeic acid (**xiv**; 38 mg) [45]. Fr. 3/2-9 was chromatographed over Sephadex LH-20 (2 cm × 50 cm), eluting with ethanol-water (90:10→10:90) to myricetin-3-*O*-β-d-glucuronopyranoside (**i**; 163 mg) [46] and 3-*O*-caffeoylquinic acid (**xv**; 14 mg) [47]. Frs. 3/3 (7 g) was chromatographed over Sephadex LH-20 (2 cm × 80 cm), eluting with ethanol-water (90:10→10:90) to give 9 fractions (frs. 3/3-1–3/3-9). Fr. 3/3-2 was separated using pTLC (solvent: toluene-EtOAc-HCOOH 5:4:1) to give kaempferol-3-*O*-α-l-rhamnopyranoside (**ix**; 37 mg) [48]. Frs. 3/3-3 and 3/3-4 were combined and chromatographed on pHPLC [Summit HPLC-system with UV-Vis detector (Dionex, Sunnyvale, CA, USA), column LiChrosorb RP-18 (10 × 250 mm, 7 μm, Merck), T 35 °C, flow rate 2 mL/min; solvent, linear gradient of 5%–40% of MeCN in 5% HCOOH/H_2_O for 60 min; detector at 350 nm) to give 12 frs. [frs. 3/3-(3-4)-1–3/3-(3-4)-12]. Fr. 3/3-(3-4)-8 (t*_R_* 38–42 min) was crystallized to give isorhamnetin-3-*O*-β-d-glucopyranoside (**vii**; 18 mg) [49]. Fr. 3/3-(3-4)-11 (t*_R_* 49–52 min) was crystallized to give isorhamnetin-3-*O*-β-d-glucuronopyranoside (**viii**; 27 mg) [42]. Frs. 3/5–3/7 were combined and chromatographed over Sephadex LH-20 (1 cm × 50 cm), eluting with ethanol-water (90:10→10:90) to give quercitrin (quercetin-3-*O*-α-l-rhamnopyranoside; **vi**; 34 mg) [50].

*Potentillin* (**x**). Off-white powder. *t*_R_ 2.122 min. UV (λ_max_) nm 220, 255. (−)ESI-MS *m*/*z* 935 [M−H]^−^. ^1^H-NMR (500 MHz, MeOH-*d*_4_) δ 7.31 (2H, c; H-2', H-6', galloyl), 6.71, 6.62, 6.54, 6.38 (each 1H, c; H-3''', H-3'''', H-3''''', H-3'''''', HHDP), 6.57 (1H, d, *J* = 3.5 Hz; H-1, α-Glc*p*), 5.60 (1H, dd, *J* = 9.1, 10.2 Hz; H-3, α-Glc*p*), 5.36 (1H, dd, *J* = 3.5, 9.1 Hz; H-2, α-Glc*p*), 5.29 (1H, dd, *J* = 6.0, 12.9 Hz; H-6_a_, α-Glc*p*), 5.23 (1H, t, *J* = 10.2 Hz; H-4, α-Glc*p*), 4.64 (1H, dd, *J* = 6.0, 10.2 Hz; H-5, α-Glc*p*), 3.82 (1H, d, *J* = 13.0 Hz; H-6_b_, α-Glc*p*). ^13^C-NMR (125 MHz, MeOH-*d*_4_) δ 171.7, 169.4, 169.0, 168.5, 168.0 (carbonyls, COO), 146.2 (C-3', C-5', galloyl), 147.0, 146.8, 146.5, 146.1 (C-6'', C-6''', C-6'''', C-6''''', HHDP), 145.9, 145.4 (2C), 145.0 (C-4'', C-4''', C-4'''', C-4''''', HHDP), 140.7 (C-4', galloyl), 137.4 (2C), 137.0, 136.8 (C-5'', C-5''', C-5'''', C-5''''', HHDP), 126.9, 126.5, 126.0, 125.7 (C-2'', C-2''', C-2'''', C-2''''', HHDP), 120.1 (C-1', galloyl), 116.7, 116.5, 115.6, 115.0 (C-1'', C-1''', C-1'''', C-1''''', HHDP), 110.1 (C-2', C-6', galloyl), 108.9, 108.4, 107.9, 107.5 (C-3'', C-3''', C-3'''', C-3''''', HHDP), 90.5 (C-1, α-Glc*p*), 75.5 (C-3, α-Glc*p*), 74.0 (C-2, α-Glc*p*), 70.7 (C-5, α-Glc*p*), 68.7 (C-4, α-Glc*p*), 63.0 (C-6, α-Glc*p*).

*Agrimonic acid A* (**xi**). Off-white powder. *t*_R_ 1.876 min. UV (λ_max_) nm 205, 274. (−)ESI-MS *m*/*z* 1103 [M−H]^−^. ^1^H-NMR (500 MHz, MeOH-*d*_4_) δ 7.32 (1H, d, *J* = 2.1 Hz; H-6', DHDG), 7.22 (1H, c; H-6'', DHDG), 6.89 (1H, d, *J* = 2.2 Hz; H-2', DHDG), 6.72, 6.68, 6.40, 6.32 (each 1H, c; H-3''', H-3'''', H-3''''', H-3'''''', HHDP), 6.55 (1H, d, *J* = 3.4 Hz; H-1, α-Glc*p*), 5.58 (1H, dd, *J* = 9.2, 10.1 Hz; H-3, α-Glc*p*), 5.34 (1H, dd, *J* = 3.4, 9.2 Hz; H-2, α-Glc*p*), 5.27 (1H, dd, *J* = 5.9, 12.9 Hz; H-6_a_, α-Glc*p*), 5.20 (1H, t, *J* = 10.1 Hz; H-4, α-Glc*p*), 4.61 (1H, dd, *J* = 5.9, 10.1 Hz; H-5, α-Glc*p*), 3.80 (1H, d, *J* = 13.1 Hz; H-6_b_, α-Glc*p*). ^13^C-NMR (125 MHz, MeOH-*d*_4_) δ 171.9, 169.9, 169.7, 169.2, 168.5, 168.2 (carbonyls, COO), 150.1 (C-3', DHDG), 147.7 (C-5', DHDG), 147.3, 147.1 (2C), 146.9 (C-6''', C-6'''', C-6''''', C-6'''''', HHDP), 145.7, 145.5, 145.2 (2C) (C-4''', C-4'''', C-4''''', C-4'''''', HHDP), 143.4 (C-5'', DHDG), 142.8 (C-3'', DHDG), 142.6 (C-4', DHDG), 141.3 (C-4'', DHDG), 138.1 (C-2'', DHDG), 137.8 (2C), 137.5 (2C) (C-5''', C-5'''', C-5''''', C-5'''''', HHDP), 127.7, 127.5, 127.1, 126.9 (C-2''', C-2'''', C-2''''', C-2'''''', HHDP), 120.9 (C-1', DHDG), 117.0 (C-1'', DHDG), 116.9, 116.8, 116.0, 115.4 (C-1''', C-1'''', C-1''''', C-1'''''', HHDP), 112.9 (C-2', DHDG), 110.8 (C-6'', DHDG), 109.3 (2C), 109.1, 108.9 (C-3''', C-3'''', C-3''''', C-3'''''', HHDP), 108.6 (C-6', DHDG), 90.9 (C-1, α-Glc*p*), 75.9 (C-3, α-Glc*p*), 74.2 (C-2, α-Glc*p*), 71.0 (C-5, α-Glc*p*), 69.2 (C-4, α-Glc*p*), 63.5 (C-6, α-Glc*p*).

*Agrimonic acid B* (**xii**). Off-white powder. *t*_R_ 1.563 min. UV (λ_max_) nm 205, 274. (−)ESI-MS *m*/*z* 1103 [M−H]^−^. ^1^H-NMR (500 MHz, MeOH-*d*_4_) δ 7.29 (1H, d, *J* = 2.1 Hz; H-6', DHDG), 7.24 (1H, c; H-6'', DHDG), 6.84 (1H, d, *J* = 2.2 Hz; H-2', DHDG), 6.74, 6.70, 6.44, 6.35 (each 1H, c; H-3''', H-3'''', H-3''''', H-3'''''', HHDP), 6.51 (1H, d, *J* = 3.4 Hz; H-1, α-Glc*p*), 5.52 (1H, dd, *J* = 9.2, 10.1 Hz; H-3, α-Glc*p*), 5.33 (1H, dd, *J* = 3.4, 9.2 Hz; H-2, α-Glc*p*), 5.21 (1H, dd, *J* = 5.9, 12.9 Hz; H-6_a_, α-Glc*p*), 5.18 (1H, t, *J* = 10.1 Hz; H-4, α-Glc*p*), 4.58 (1H, dd, *J* = 5.9, 10.1 Hz; H-5, α-Glc*p*), 3.67 (1H, d, *J* = 13.1 Hz; H-6_b_, α-Glc*p*). ^13^C-NMR (125 MHz, MeOH-*d*_4_) δ 171.7, 170.3, 169.9, 169.2, 168.6, 168.0 (carbonyls, COO), 149.9 (C-3'', DHDG), 147.5 (C-5', DHDG), 147.1, 147.2 (2C), 146.7 (C-6''', C-6'''', C-6''''', C-6'''''', HHDP), 145.8, 145.4 (2C), 145.1 (C-4''', C-4'''', C-4''''', C-4'''''', HHDP), 143.8 (C-5'', DHDG), 143.1 (C-3', DHDG), 142.8 (C-4'', DHDG), 141.5 (C-4', DHDG), 138.0, 137.9, 137.6, 137.4 (C-5''', C-5'''', C-5''''', C-5'''''', HHDP), 136.9 (C-2′, DHDG), 127.8, 127.5, 127.0, 126.7 (C-2''', C-2'''', C-2''''', C-2'''''', HHDP), 121.4 (C-1', DHDG), 117.2 (C-1'', DHDG), 116.6, 116.7, 115.9, 115.2 (C-1''', C-1'''', C-1''''', C-1'''''', HHDP), 112.4 (C-2'', DHDG), 110.7 (C-6'', DHDG), 109.4, 109.2, 109.0, 108.7 (C-3''', C-3'''', C-3''''', C-3'''''', HHDP), 108.4 (C-6′, DHDG), 90.5 (C-1, α-Glc*p*), 76.2 (C-3, α-Glc*p*), 74.6 (C-2, α-Glc*p*), 71.5 (C-5, α-Glc*p*), 68.8 (C-4, α-Glc*p*), 63.2 (C-6, α-Glc*p*).

*Agrimoniin* (**xiii**). Off-white powder. *t*_R_ 2.063 min. UV (λ_max_) nm 230, 268. (−)ESI-MS *m*/*z* 1869 [M−H]^−^. ^1^H-NMR (500 MHz, MeOH-*d*_4_) δ 7.34 (1H, d, *J* = 2.1 Hz; H-6'', DHDG), 7.20 (1H, c; H-6''', DHDG), 6.91 (1H, d, *J* = 2.1 Hz; H-2'', DHDG), 6.73, 6.70, 6.65, 6.62, 6.60, 6.58, 6.51, 6.46 (each 1H, c; H-3'''', H-3''''', H-3'''''', H-3''''''', H-3'''''''', H-3''''''''', H-3'''''''''', H-3''''''''''', HHDP), 6.56 (1H, d, *J* = 4.0 Hz; H-1, α-Glc*p*-1), 6.51 (1H, d, *J* = 4.0 Hz; H-1', α-Glc*p*-2), 5.52 (1H, dd, *J* = 9.6, 10.2 Hz; H-3', α-Glc*p*-2), 5.48 (1H, dd, *J* = 9.6, 10.2 Hz; H-3, α-Glc*p*-1), 5.38 (1H, dd, *J* = 4.0, 9.5 Hz; H-2', α-Glc*p*-2), 5.32 (1H, dd, *J* = 4.0, 9.5 Hz; H-2, α-Glc*p*-1), 5.29 (1H, dd, *J* = 6.1, 13.0 Hz; H-6'_a_, α-Glc*p*-2), 5.22 (1H, dd, *J* = 6.0, 13.0 Hz; H-6_a_, α-Glc*p*-1), 5.20 (1H, t, *J* = 9.5 Hz; H-4', α-Glc*p*-2), 5.14 (1H, t, *J* = 9.5 Hz; H-4, α-Glc*p*-1), 4.61 (1H, dd, *J* = 6.1, 10.2 Hz; H-5', α-Glc*p*-2), 4.47 (1H, dd, *J* = 6.1, 10.2 Hz; H-5, α-Glc*p*-1), 3.70 (1H, d, *J* = 13.6 Hz; H-6'_b_, α-Glc*p*-2), 3.65 (1H, d, *J* = 13.6 Hz; H-6_b_, α-Glc*p*-1). ^13^C-NMR (125 MHz, MeOH-*d*_4_) δ 171.4, 171.0, 169.7 (2C), 169.5, 169.3, 169.0 (2C), 166.6, 166.3 (carbonyls, COO), 150.1 (C-3'', DHDG), 147.9 (C-5'', DHDG), 147.5 (4C), 147.1, 146.8 (2C), 146.5 (C-6'''', C-6''''', C-6'''''', C-6''''''', C-6'''''''', C-6''''''''', C-6'''''''''', C-6''''''''''', HHDP), 146.0, 145.8, 145.5 (3C), 145.1, 144.7, 144.3 (C-4'''', C-4''''', C-4'''''', C-4''''''', C-4'''''''', C-4''''''''' C-4'''''''''', C-4''''''''''', HHDP), 143.4 (C-5''', DHDG), 142.8 (C-3''', DHDG), 142.6 (C-4'', DHDG), 141.3 (C-4''', DHDG), 138.1 (C-2''', DHDG), 137.6 (2C), 137.3, 136.8, 136.5 (3C), 136.2 (C-5'''', C-5''''', C-5'''''', C-5''''''', C-5'''''''', C-5''''''''', C-5'''''''''', C-5''''''''''', HHDP), 126.7, 126.4, 126.2, 126.0 (2C), 125.7, 125.4 (2C) (C-2'''', C-2''''', C-2'''''', C-2''''''', C-2'''''''', C-2''''''''', C-2'''''''''', C-2''''''''''', HHDP), 120.9 (C-1'', DHDG), 117.0 (C-1''', DHDG), 116.7, 116.5 (2C), 116.0, 115.8, 115.6 (2C), 115.3 (C-1'''', C-1''''', C-1'''''', C-1''''''', C-1'''''''', C-1''''''''', C-1'''''''''', C-1''''''''''', HHDP), 112.9 (C-2'', DHDG), 110.8 (C-6''', DHDG), 109.1, 108.7 (4C), 108.5, 108.3 (2C) (C-3'''', C-3''''', C-3'''''', C-3''''''', C-3'''''''', C-3''''''''', C-3'''''''''', C-3''''''''''', HHDP), 108.0 (C-6'', DHDG), 90.7 (C-1, α-Glc*p*-1), 91.2 (C-1', α-Glc*p*-2), 76.0 (C-3', α-Glc*p*-2), 75.6 (C-3, α-Glc*p*-1), 74.4 (C-2, α-Glc*p*-1), 74.1 (C-2', α-Glc*p*-2), 71.4 (C-5', α-Glc*p*-2), 71.0 (C-5, α-Glc*p*-1), 68.9 (C-4, α-Glc*p*-1), 68.5 (C-4', α-Glc*p*-2), 63.3 (C-6, α-Glc*p*-1), 63.1 (C-6', α-Glc*p*-2).

### 3.4. HPLC Apparatus

The HPLC analyses were performed using the MiliChrom A-02 system (Econova, Novosibirsk, Russia) equipped with a dual low-pressure gradient pump with vacuum degasser, an autosampler, a column compartment, and a UV-Vis detector.

### 3.5. MC-RP-HPLC-UV Conditions

Separation was carried out with ProntoSIL-120-5-C18 AQ analytical column (1 mm × 60 mm × 5 μm; Metrohm AG; Herisau, Switzerland). Column temperature was maintained at 35 °C. Elution was conducted using eluent A (0.2 М LiClO_4_ in 0.006 M HClO_4_) and eluent B (acetonitrile) with a six-step gradient as follows: 0–1.25 min 11%–18% B, 1.25–2.25 min 18% B, 2.25–2.5 min 18%–20% B, 2.5–3.0 min 20%–25% B, 3.0–4.0 min 25% B, 4.0–5.0 min 25%–100% B. The flow rate was 0.6 mL·min^−1^. Then, 1 μL of each sample was introduced by the autosampler to the column. The column was equilibrated 1 min between injections. UV spectra were recorded in the range of 200–400 nm. Chromatogramms were acquired at 270 nm.

### 3.6. Preparation of Standard Solutions for Quantification

Stock solutions of standards were made by accurately weighing 3 mg of caffeic acid, myricetin-3-*O*-glucuronide, agrimoniin, ellagic acid, miquelianin, isorhamnetin-3-*O*-glucuronide, kaempferol-3-*O*-rhamnoside and dissolving it in 1 mL of methanol in a volumetric flask. The appropriate amounts of stock solutions were diluted with methanol in order to obtain standard solutions containing 2–2500 μg/mL. As all the compounds used for quantification were well-separated in experiment conditions mixtures of standards were analyzed. Prepared solutions were stored at 4 °C for no more than 72 h.

### 3.7. Sample Preparation for Quantification

An accurately weighted, dried, and powdered *P. anserina* plant sample (100 mg) was placed in a conical flask. Then 4 mL of 60% acetone was added and the mixture was weighted. The sample was then extracted for 30 min at 40 °C in ultrasonic device UZV-2.8 (Sapfire, Moscow, Russia) with an ultrasound power of 100 W and frequency of 35 kHz, equipped with a temperature controller and a digital timer. After cooling, the flask weight was reduced to initial sign, and the resultant extract was filtered through a 0.22-μm PTFE syringe filter before injection into the HPLC system for analysis.

For preparation of decoction, an accurately weighted, dried, and powdered *P. anserina* plant sample (1 g) was placed in a conical flask. Then 20 mL of deionised water was added and the mixture was weighted. The sample was then boiled in a water bath for 30 min. After 10 min of cooling, the flask weight was reduced to initial sign, and the resultant extract was filtered through a 0.22-μm PTFE syringe filter before injection into the HPLC system for analysis.

For preparation of infusion, an accurately weighted, dried, and powdered *P. anserina* plant sample (1 g) was placed in a conical flask. Then 20 mL of boiled deionised water was added and the mixture was weighted. The sample was then stirred for 40 min, the flask weight was reduced to initial sign, and the resultant extract was filtered through a 0.22-μm PTFE syringe filter before injection into the HPLC system for analysis. Samples of liquid extract and tincture were filtered through a 0.22-μm PTFE syringe filter before injection into the HPLC system for analysis.

For preparation of dry extract solution, an accurately weighted dry extract of *P. anserina* (10 mg) was placed in an Eppendorf tube, 1 mL of 60% ethanol was added, and the mixture was weighted. Then the sample was extracted in an ultrasonic bath for 10 min at 40 °C. After cooling, the tube weight was reduced to initial sign, and the resultant extract was filtered through a 0.22-μm PTFE syringe filter before injection into the HPLC system for analysis.

### 3.8. Validation

For validation of the analytical method, the guidelines established by the International Conference on the Harmonization of Technical Requirements for the Registration of Pharmaceuticals for Human Use (ICH) were employed [51]. The linearity of the method was studied by injecting five known concentrations of the standard compounds in the defined range. Results from each analysis were averaged and subjected to regression analysis. Limits of detection (LOD) and quantification (LOQ) were determined using the equations LOD = 3.3 × *S_YX_*/*a* and LOQ = 10 × *S_YX_*/*a*, respectively, where *S_YX_* is a standard deviation of the response (*Y* intercept) and *a* is a slope of calibration curve. The stability test was performed with one sample solution, which was stored at room temperature and analyzed at 0, 2, 4, 8, 12, and 24 h. The precision of the analytical method was evaluated by intra-day, inter-day, and repeatability test. Intra-day assay was determined by assaying three different concentrations of each compound among the linearity concentrations during the same day, and inter-day assay was analyzed using the same concentrations for intra-day precision on three different days (interval of 1 day) in the same laboratory. The repeatability test of the sample was performed on quintuple experiments of three different concentrations. For analysis of recovery data, the appropriate amounts of the powdered sample of seven phenolic compounds were weighted and spiked with a known amount of each reference compound and then analyzed. Each sample was analyzed in quintuple.

### 3.9. Statistical Analysis

Statistical analyses were performed using a one-way analysis of variance (ANOVA), and the significance of the mean difference was determined by Duncan’s multiple range test. Differences at *p* < 0.05 were considered statistically significant. The results are presented as mean values ± SD (standard deviations) of the three replicates.

## 4. Conclusions

At the present study, a new rapid microcolumn RP-HPLC-UV method has been developed to simultaneously determine seven major phenolic compounds in the *Potentilla anserina* L. herb. The method showed a good linearity, precision, and accuracy, so it was suitable for quality control of the commercial samples of *P. anserina* L. herb. Ultrasound-assisted extraction was successfully employed to optimize the extraction, and several experimental parameters have been evaluated. The best combination of response functions was 60% acetone, a liquid-to-solid ratio of 40:1, and an extraction of 30 min at 40 °C under ultrasound irradiation of 100 W. In addition, 12 samples collected in different Siberian regions and two commercial herbs and preparations have been analyzed. The results warrant further discussion of the appropriate quality control of *P. anserina* pharmaceuticals.
